# COVID-19 masks increase the influence of face recognition algorithm decisions on human decisions in unfamiliar face matching

**DOI:** 10.1371/journal.pone.0277625

**Published:** 2022-11-21

**Authors:** Daniela Barragan, John J. Howard, Laura R. Rabbitt, Yevgeniy B. Sirotin

**Affiliations:** 1 Identity and Data Sciences Laboratory (IDSL), SAIC, Reston, VA, United States of America; 2 Maryland Test Facility (MdTF), Upper Marlboro, MD, United States of America; Cleveland State University, UNITED STATES

## Abstract

Face masks, recently adopted to reduce the spread of COVID-19, have had the unintended consequence of increasing the difficulty of face recognition. In security applications, face recognition algorithms are used to identify individuals and present results for human review. This combination of human and algorithm capabilities, known as human-algorithm teaming, is intended to improve total system performance. However, prior work has shown that human judgments of face pair similarity-confidence can be biased by an algorithm’s decision even in the case of an error by that algorithm. This can reduce team effectiveness, particularly for difficult face pairs. We conducted two studies to examine whether face masks, now routinely present in security applications, impact the degree to which this cognitive bias is experienced by humans. We first compared the influence of algorithm’s decisions on human similarity-confidence ratings in the presence and absence of face masks and found that face masks more than doubled the influence of algorithm decisions on human similarity-confidence ratings. We then investigated if this increase in cognitive bias was dependent on perceived algorithm accuracy by also presenting algorithm accuracy rates in the presence of face masks. We found that making humans aware of the potential for algorithm errors mitigated the increase in cognitive bias due to face masks. Our findings suggest that humans reviewing face recognition algorithm decisions should be made aware of the potential for algorithm errors to improve human-algorithm team performance.

## Introduction

The COVID-19 pandemic has imposed many new requirements, such as mask usage to limit the spread of the virus [1]. However, masks have introduced unique challenges to security scenarios where identity verification is a critical component. Establishing the identity of unfamiliar individuals using facial characteristics is a task performed frequently by human security officers that have to match unidentified live faces or photographs to reference photos linked with known identities such as those found on drivers’ licenses or passports. To facilitate the process of matching unfamiliar individual’s identity in security settings, face recognition algorithms (algorithms) are deployed in security settings, creating human-algorithm teams. In these teams, an algorithm arrives at an identity decision and reports it to a human operator for confirmation. However, the presence of face masks increases the difficulty of matching faces for both humans and algorithms. This research studies the effectiveness of these teams in the presence of face masks and highlights critical weaknesses that may be impacting everyday performance of these human algorithm teams.

Traditionally, humans have performed identity verification tasks because humans have evolutionary features dedicated to face recognition [2] which are well studied in laboratory environments [3–5]. Though humans excel at matching familiar faces, matching unfamiliar faces, as is the case in security applications, is a difficult task for most humans [6]; however, there are those with exceptional abilities, known as super-recognizers [7]. Human unfamiliar face matching performance has been well studied and has been found to be affected by numerous factors including image resolution [8], pose [9], illumination [10], and age of the photographs [6]. Even glasses have been shown to decrease face recognition and face matching performance [11, 12].

Specialized computer programs called face recognition algorithms (algorithms) have been developed to assist humans with unfamiliar face matching. Performance benchmarks published by the U.S. National Institute of Standards and Technology (NIST) show that algorithm errors decreased from 5% in 2010 to just 0.2% in 2018 [13]. This advance in algorithm performance has spurred increased automation of face recognition in security operations, such as in aviation [14], immigration [15] and law enforcement [16, 17]. Because even the best algorithms still make mistakes, algorithm outcomes are reviewed by humans to make a final identity determination. This combination of human abilities with algorithms is referred to as human-algorithm teaming.

With increasing adoption of algorithms in critical applications, such as security, it is important to understand the effectiveness of human oversight of algorithm decisions [18]. Ideally, the performance of human-algorithm teams should exceed that of either humans or algorithms working in isolation. Phillips and colleagues showed that averaging algorithm with independently made, human face similarity judgments can lead to higher face matching accuracy [19], similar to that achieved by pooling multiple independent human decisions [20]. However, humans do not make independent face similarity judgments when they review algorithm identity decisions. Indeed, Howard and colleagues [21] recently showed that prior algorithm decisions, whether correct or erroneous, cognitively bias human face similarity judgments, especially for difficult face pairs, potentially limiting the total accuracy of a human-algorithm team in a manner similar to “herding behavior” in social psychology [22, 23].

Recent work has also examined the impact of face masks on unfamiliar face matching performance. The occlusion of facial characteristics by face masks disrupts human ability to process faces [24] and reduces algorithm performance [25]. Using a signal detection paradigm, Carragher and Hancock examined both human and algorithm accuracy in the presence of face masks. They showed that human face sensitivity was reduced when faces in one or both photographs to be matched were occluded by face masks [26] and that algorithm accuracy was reduced when one of the faces was masked, concluding that “Identification decisions for masked faces should be treated with caution.” Nonetheless, algorithm performance in the presence of face masks have been shown to achieve error rates in the range of 1–3%, making them viable for consideration in security applications [25]. This raises important questions regarding the performance of human-algorithm teams in the presence of face masks.

We conducted two studies to investigate how prior face identity decisions influence subsequent human judgments in the context of a human-algorithm team performing a face matching task. We replicated and adapted the face matching task developed by Howard and colleagues [21]. In this task, reviewers reviewed a pair of faces presented with a same identity or different identity decision from an algorithm and then determine if the pair shows the same person or different people using a 7-point similarity-confidence scale.

**Fig 1:**
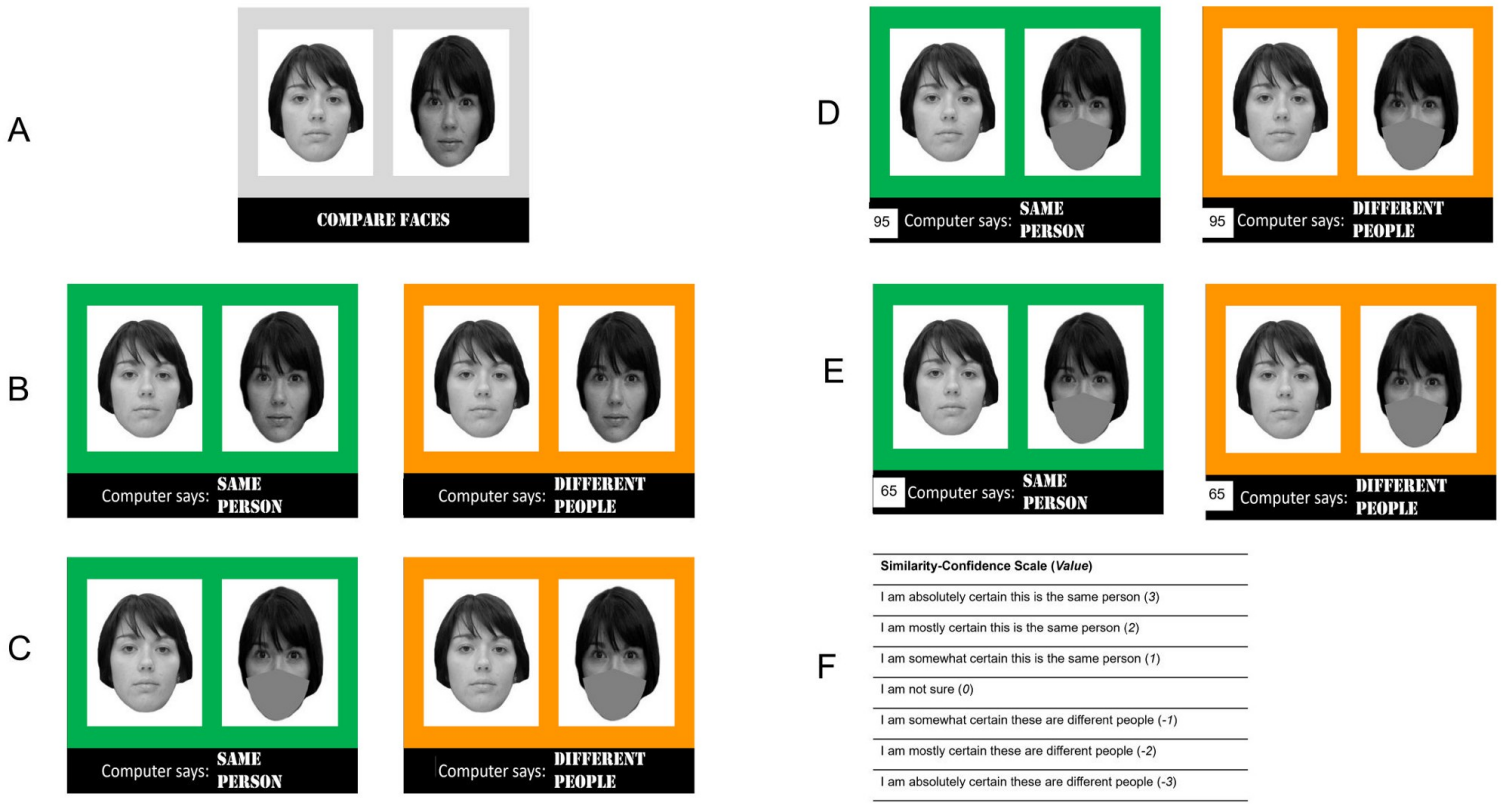
Example of face pair stimuli (from the Glasgow Face Matching Task (GFMT) [3]) and experimental conditions used. After viewing each stimulus, reviewers were asked to rate the similarity of the faces. **A**. Study 1 and 2 control condition. Reviewers were presented with pairs of faces without any additional information. **B**. Study 1 no mask condition. Face pairs were presented with along with prior algorithm identity decisions communicated by text and color (“SAME PERSON”/green and “DIFFERENT PEOPLE”/orange). **C**. Study 1 mask condition. The right face image in each pair was digitally altered with a digital neutral gray mask covering the lower portion of the face. Prior identity decisions were presented as in B. **D** and **E**. Study 2 presentation of algorithm accuracy information. The number in the lower left of each face pair reminded reviewers of algorithm’s accuracy as explained at the beginning of the task. Prior identity decisions were presented as in B and C. **F**. Similarity-confidence scale reviewers used to rate each face pair in Study 1 and Study 2.

Study 1 focused on face matching performance, with and without face masks alongside an algorithm decision (i.e., a computer says “SAME PERSON” or “DIFFERENT PEOPLE”; see Fig 1B and 1C). Study 2 examined the effects of masks and algorithm accuracy rates on reviewer similarity-confidence ratings. Reviewers were informed that they were reviewing decisions from an algorithm that was labeled as 65% or 95% accurate (see Fig 1D and 1E). Study 1 and Study 2 also each included a control condition where algorithm decisions, algorithm accuracy information, and masks were absent (see Fig 1A). We also asked reviewers to rate their level of trust in the identity decisions presented from the algorithm (or themselves in the control condition) prior to performing the face matching task (Studies 1 and 2) and after the face matching task (Study 2). Trust in autonomous systems, such as an algorithm generating face matching decisions, has been shown to affect human performance, conformity, and reliance on the system [27]. Thus, including this question both before and after the matching task in Study 2 allowed us to assess if different algorithm accuracy rates modulated reviewers’ trust levels.

Our results highlight the challenges associated with human-algorithm teaming, particularly when less face information is available due to masks, and how humans may rely more heavily on an algorithm’s decision when they themselves lack information. We found that masks not only decreased accuracy, but also shifted human similarity-confidence rating responses in the direction of the algorithm decision to a greater degree than when masks were absent. Additionally, when masks and algorithm accuracy rates were combined, we observed responses shifted differently depending on the algorithm accuracy rate. We show not only how the masks influence human decisions, but how algorithm information can also influence decision making and trust, highlighting the importance of testing human-algorithm teams with different challenges and how informing users of biometric systems can influence decision making.

## Materials and methods

All consent materials and testing protocols were approved by an Institutional Review Board (New England IRB) under protocol “Development and Evaluation of Enhanced Screening” number 120180237. Reviewers for the studies were recruited from the Maryland, Northern Virginia, and D.C. areas. Prior to participation, all reviewers performed multiple consent activities. The first consent activity was group consent, where a researcher provided an overview to all reviewers about the purpose of the study and experimental protocols. The second consent activity was individual consent, where reviewers were provided an opportunity to ask questions about the consent form to a researcher in a private room. Reviewers acknowledged their understanding and consent to participate in the study by signing the consent form.

Reviewers were recruited as part of separate biometrics tests at the Maryland Test Facility. Reviewers self reported sex, age, and race prior to the study during screening procedures (details in S1 File). During testing, reviewers first performed biometric transactions using a diverse set of technologies as part of an unrelated experiment. Reviewers were then tasked with completing a face matching task administered on iPads (Apple; Cupertino, CA) using survey software (Qualtrics; Provo, UT). Reviewers did not have a time-limit for completing the task, but most finished the full survey within 15 minutes. Response times to each question ranged from 10 to 14 seconds on average (additional information on response times are described in S1 File). An analysis of response times and performance was not performed.

### Core face matching task

The face matching tasks were modified based on Howard et al. (2020) [21]. Briefly, all task variants consisted of 14 face pairs: eight face pairs from the Glasgow Face Matching Task-short version (GFMT-short [3]), four face pairs from the Multiple Encounters Dataset (MEDS [28]), and two pairs of celebrity faces. The celebrity faces were widely known figures in the U.S. and served as an attentional question; reviewers that did not respond correctly to one or both of the celebrity face pairs were excluded from analysis. Face pairs from the GFMT-short were selected based on similarity scores from a commercial face recognition algorithm; six face pairs with the highest similarity scores for same and different identities were selected. MEDS face pairs were selected using the same criteria and then formatted to match the style of GFMT stimuli (i.e., images converted to gray scale and cropped). The similarity scores were obtained from an algorithm representative of the types of algorithms deployed at security checkpoints; no comparison of algorithms were included in the studies. Face pairs selected from the GFMT-short and MEDS were balanced for gender.

During the task, reviewers had to rate the similarity of each face pair using a 7-point similarity-confidence scale based on their judgement of the face pair and prior identity decisions (“SAME PERSON” or “DIFFERENT PEOPLE”) from a fictional face recognition algorithm. All algorithm prior identity decisions presented during the task were random. Algorithm decisions were presented simultaneously with each face pair to simulate the runtime of actual face recognition algorithms. In real-world applications of automated face recognition algorithms, matching occurs so quickly that the results seem instantaneous to users.

Each reviewer was randomly assigned to one of two survey variants, nested within each experimental condition, where a given face pair was presented with either “SAME PERSON” or “DIFFERENT PEOPLE” algorithm decision. Within each variant, half of the face pairs were presented with “SAME PERSON” and half with “DIFFERENT PEOPLE” algorithm decisions. Some reviewers in each study were randomly assigned to a control condition which did not include any prior identity information. This control condition served as a reference for experimental manipulations and confirmed that changes in test context across studies did not alter general face matching performance. Prior to beginning the task, reviewers were asked to rate their trust in either the algorithm, if assigned to an experimental condition, or themselves, if assigned to the control condition (S1 and S4 Figs in S1 File). Additional methods are presented in (S1 File).

### Study 1

A total of 150 reviewers were included in the analysis (details in S1 File). This experiment examined the impact of face mask on human review. Two experimental face matching conditions were tested: each face pair was presented with prior algorithm decisions either without face masks or with digitally applied face masks (S1 File). Prior to beginning the task, reviewers were asked to rate their trust in either the algorithm (if assigned to the mask or no mask condition) or themselves (if assigned to the control condition). Reviewers’ responses to the trust question did not differ across conditions indicating they had similar trust levels for themselves and algorithms (S1 Fig in S1 File).

### Study 2

A total of 497 reviewers were included in the analysis (details in S1 File). This experiment examined the impact of information about algorithm accuracy on human review. Prior to starting the face matching task, reviewers were informed regarding the accuracy of the computer algorithm. Two experimental conditions were tested: reviewers were told that the computer was correct 65% of the time or 95% of the time. To ensure that reviewers understood and remembered this information, they were asked answer a multiple-choice questions about the presented algorithm accuracy prior to starting the task. Reviewers that failed to select the correct accuracy were excluded from analysis. Both experimental conditions included face pairs with face masks as in the with-masks condition of Study 1. Additionally, algorithm accuracy information (65% or 95%) was presented numerically next to each face pair as a reminder. Following the face matching tasks, reviewers in Study 2 were asked to rate their trust in either the algorithm, if assigned to an experimental condition, or themselves in the control condition a second time (S6 Fig in S1 File).

### Data analyses

Data analyses utilized signal detection theory (SDT) to calculate the area under the curve (AUC) and create receiver operating characteristic (ROC) curves [29]. Relevant data analyses methods, including formulas to calculate true positive rate (TPR), false positive rate (FPR), and overall accuracy are described in S1 File. Additional discussion about other SDT metrics, such as sensitivity and the criterion can also be found in S1 File.

## Results

### Study 1—Effects of masks and algorithm decisions on unfamiliar face matching

Study 1 examined the influence of face masks on human reviewer accuracy and on the cognitive biases introduced by prior algorithm identity decisions (i.e., the shifts in similarity-confidence ratings observed when the two faces were labeled as “SAME PERSON” versus as “DIFFERENT PEOPLE” by the algorithm; see Fig 1B and 1C). Reviewers (*n* = 150) were assigned to one of three face matching conditions: control, no mask, and mask. In the control condition, reviewers matched face pairs without prior algorithm decisions and without masks (Fig 1A). This condition served to ensure that the test was yielding results consistent with other evaluations of face perception. In the no mask condition, reviewers matched face pairs together with prior algorithm identity decisions (Fig 1B) similar to [21]. In the mask condition, reviewers matched the same face pairs with algorithm identity decisions, but the right face of each pair was obscured by digital mask (Fig 1C). The goal of this condition was to replicate prior work showing reduce face matching performance in the presence of face masks and to determine whether algorithm decisions influenced reviewer responses more in the presence of face masks. Each reviewer completed a single test condition with 12 face pairs. Reviewers responded to each face pair by selecting an option on a seven point similarity-confidence scale ranging from “I am absolutely certain these are different people” to “I am absolutely certain this is the same person” (full similarity-confidence scale displayed in Fig 1F).

#### Face masks reduce face matching accuracy

We analyzed face matching similarity-confidence ratings by thresholding similarity-confidence values such that responses of 1 and greater (*θ* = 0.5) on the similarity-confidence scale (corresponding to “I am somewhat certain this is the same person”) were set as indicating a review outcome confirming that the face pair belonged to the same person. On the other hand, responses of 0 and below on the scale (corresponding to “I am not sure”) were set as indicating that the face pair belonged to different people. These same/different outcomes were compared to the true identities of each pair.

Overall face matching accuracy was consistent with [21] and with known common norms [3]. Table 1 presents accuracy (% Correct) for all experimental conditions. Accuracy in the control condition, 77%, was comparable to prior work (Table 1; compare with 74% in [21]). Without masks, accuracy varied little based on algorithm decision, ranging between 79% and 81% (Table 1; compare with 73%-75% in [21]). The similarity of accuracy rates between this study and prior work highlight the repeatability of this face matching task. With masks, however, accuracy was markedly lower, ranging between 63% and 70%.

**Table 1:** Face matching performance as a function of test condition for Study 1 (threshold = 0.5).

Condition	Mask/No Mask	Algorithm Decision	n	% Correct (95% CI)	FPR (95% CI)	TPR (95% CI)	Sensitivity—*d’* (95% CI)	Criterion—*c* (95% CI)
Control	No Mask	None	49	0.77 (0.74–0.81)	0.14 (0.10–0.19)	0.69 (0.63–0.75)	1.56 (1.29–1.82)	0.29 (0.14–0.42)
Algorithm	No Mask	Different	51	0.79 (0.74–0.85)	0.16 (0.10–0.23)	0.75 (0.68–0.83)	1.66 (1.24–2.03)	0.15 (-0.03–0.32)
Algorithm	No Mask	Same	51	0.81 (0.76–0.85)	0.21 (0.14–0.28)	0.82 (0.76–0.89)	1.74 (1.38–2.06)	-0.06 (-0.25–0.13)
Algorithm	Mask	Different	50	0.70 (0.64–0.75)	0.13 (0.08–0.18)	0.52 (0.44–0.60)	1.19 (0.87–1.50)	0.55 (0.38–0.71)
Algorithm	Mask	Same	50	0.63 (0.57–0.69)	0.33 (0.24–0.43)	0.59 (0.51–0.67)	0.67 (0.31–1.02)	0.10 (-0.07–0.26)

To determine whether reviewer accuracy was significantly altered by masks (mask/no mask) and whether accuracy changed based the algorithm’s prior identity decisions (same/different), we conducted two-way repeated measures analysis of variance (ANOVA). Because algorithm decisions were absent in the control condition, it was excluded from the ANOVA. We found that face masks had a significant effect on overall accuracy (*F*(1, 100) = 24.06, *p* < .0001) indicating that masks decrease overall accuracy when matching a masked face to a non-masked face. However, prior algorithm decisions did not impact overall accuracy significantly (*F*(1, 100) = 0.92, *p* = 0.34) and the algorithm decision-mask interaction was not significant (*F*(1, 100) = 2.09, *p* = 0.15). In line with prior literature, this indicates that masks increased the difficulty of the face matching task significantly, reducing the accuracy of human face matching decisions [26].

We also examined if the presence of masks reduced similarity-confidence ratings. An ANOVA examining the effect of masks on the absolute values of similarity-confidence ratings did not find a significant effect (*F*(2, 147) = 0.60, *p* = 0.55). Reviewers assigned to the mask condition had equal mean absolute values of similarity-confidence (*μ* = 1.88) as those assigned to the no mask condition (*μ* = 1.88). Thus, reviewers’ similarity-confidence decisions were not systematically reduced in the presence of face masks, despite significant reductions in accuracy.

#### Masks increase the influence of algorithm decisions

To understand how algorithm decisions shift reviewer similarity-confidence ratings across different face pairs, we compared average similarity-confidence ratings as a function of algorithm decision (“SAME PERSON” or “DIFFERENT PEOPLE”) for each condition (mask versus no mask; control condition excluded from analysis) for each of the 12 face pairs. In the no mask condition, shifts in average similarity-confidence rating (Δ*μ* = *μ*_*different*_ − *μ*_*same*_) were significant for 1 of the 12 face pairs as compared with 4 of the 12 pairs in the mask condition (S3 Fig in S1 File). Across the 12 face pairs, confidence shifts were statistically larger in the mask condition as compared with the no mask condition (*t*(11) = 2.63, *p* < 0.05). These shifts in reviewer similarity-confidence ratings across the face pairs would be reflected in their sensitivity and cognitive biases when making same/different judgments.

We computed the signal detection theory metrics of sensitivity (*d*′) and criterion (*c*) [29] for each test condition. Sensitivity (*d*′) measures how well reviewers can distinguish between “SAME PERSON” and “DIFFERENT PEOPLE” face pairs [21, 26]. The internal psychological criterion (*c*) measures the degree of sensory information is needed to produce a given similarity-confidence rating. A higher criterion *c* indicates that more sensory information is needed to produce a given rating. Table 1 shows the sensitivity and criterion for each algorithm decision and mask condition based on average true positive rate (TPR) and false positive rate (FPR) measures as well as 95% confidence intervals (95% CI) calculated for each metric using bootstrap re-sampling.

Algorithm decisions altered reviewers’ criterion (*c*) for making similarity judgments. The shift in the criterion for same and different algorithm decisions (Δ*c* = *c*_*different*_ − *c*_*same*_) quantifies the degree of reviewers’ cognitive bias introduced by algorithm information [21]. Without face masks, cognitive bias was Δ*c* = 0.21 (Table 1), in line with prior work (0.22 in [21]). When masks were present, reviewer cognitive bias more than doubled to Δ*c* = 0.45. This shows that face masks increase the impact of algorithm decisions on reviewer face matching decisions.

**Fig 2:**
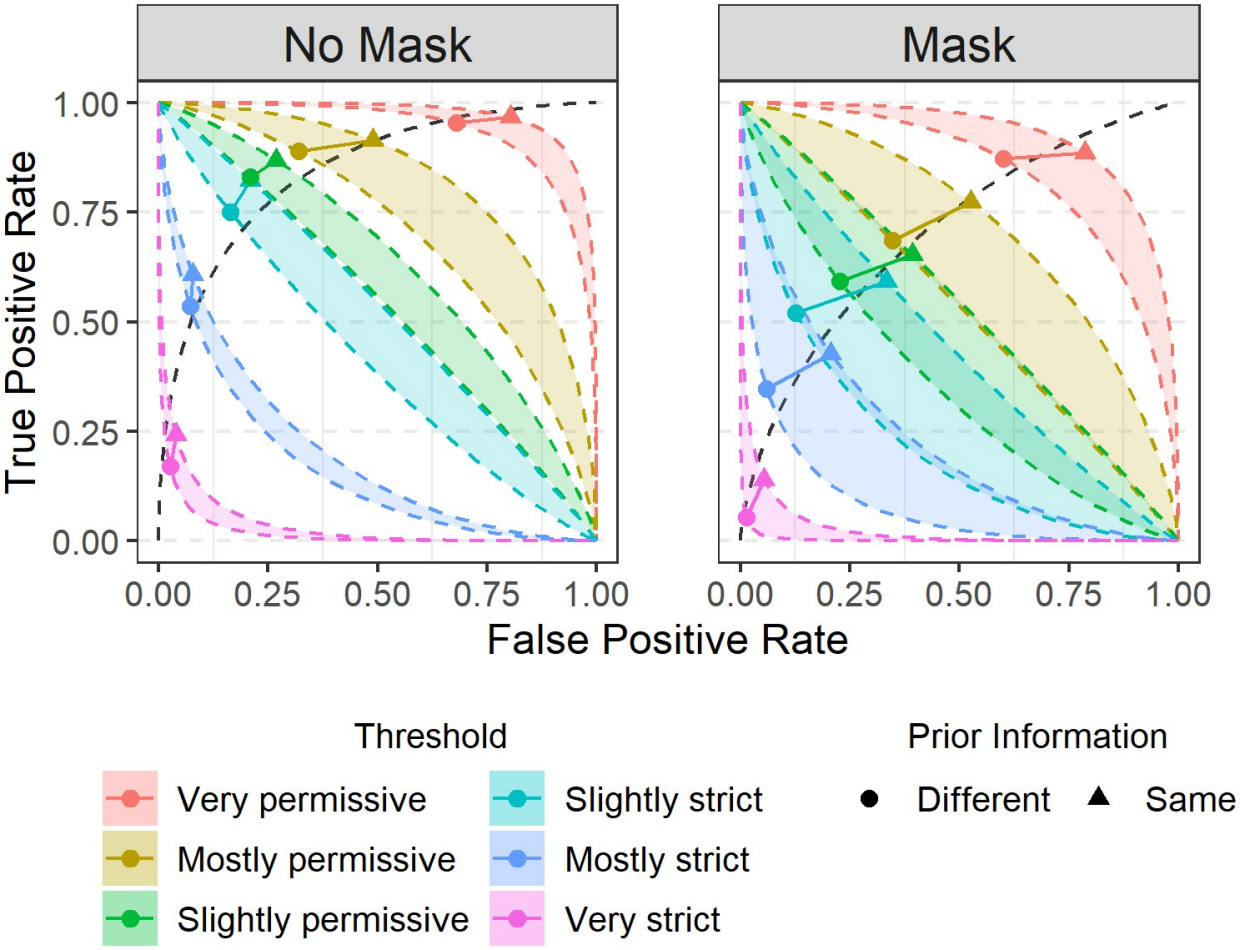
Isobias bands defined by shifts in responses by algorithm decisions across task conditions. Points denote measured TPR/FPR values computed as a function of decision threshold (legend). Lines join points associated with same/different algorithm decisions. Colored regions, isobias bands, are formed by curves in ROC space corresponding to constant criterion values for same and different algorithm decisions at each decision threshold (legend). Isobias band overlap indicates a that the shift in the cognitive criterion based on algorithm outcome is comparable to a full step along the decision confidence scale. There is very little overlap of isobands in the No Mask condition (left panel) whereas there are many regions that overlap in the Mask condition (right panel).

Fig 2 illustrates how masks increased the impact of algorithm decisions on reviewer similarity-confidence ratings. The figure shows how algorithm decisions alter the reviewers’ receiver operating characteristic (ROC) curves, which depict TPR as a function of FPR across the full set of similarity-confidence thresholds ranging from very permissive (*θ* = −2.5) to very strict (*θ* = 2.5) relative to the similarity-confidence scale (Fig 1F). As in our prior work, the effects of algorithm decisions cognitively biased the reviewers, reliably shifting reviewer criterion values [21] in both conditions which can be read out as shifts of TPR and FPR values along the ROC curve (dashed line). The impact of these decisions is visualized by the shaded isobias bands, which define the span of criterion values measured for “SAME PERSON” and “DIFFERENT PEOPLE” algorithm decisions at a given threshold. Without masks, higher-confidence decisions were only modestly affected as indicated by non-overlapping isobias bands and even the slightly permissive/strict isobias bands just osculated (Fig 2, No Mask). With masks, however, the isobias bands overlapped extensively, even at strict decision thresholds such that all but the highest similarity-confidence decisions were now affected. Thus, in the presence of face masks, algorithm decisions may shift responses on the face similarity scale even at higher levels of similarity-confidence (e.g. from “I am somewhat certain this is the same person” to “I am mostly certain this is the same person”).

### Study 2—Effects of masks and algorithm accuracy rates on unfamiliar face matching

We wanted to determine whether the increase in the cognitive biases introduced by face masks could be mitigated. Prior work indicated that most reviewers trust algorithms to make identity decisions as much as other people and that they distrust other people more than algorithms [21]. We designed Study 2 to determine whether providing explicit information about algorithm accuracy, highlighting the fact that algorithms make errors, would temper this trust and mitigate increased reviewer cognitive biases in the presence of face masks.

Study 2 provided reviewers algorithm accuracy rates in the presence of face masks (see Fig 1D and 1E). We presented reviewers with the same masked face pairs, but now included additional information about algorithm accuracy rates. Reviewers were assigned to one of three conditions. In the first condition, reviewers were told that the algorithm’s decisions were correct 65% of the time and reviewed face pairs with a mask (65-algorithm condition). In the second condition, reviewers were informed that the algorithm’s decisions were correct 95% of the time and reviewed face pairs with a mask (95-algorithm condition). The third condition was the control condition, which did not contain any algorithm decisions or masks. All accuracy rates were fictitious and algorithm decisions were actually correct 50% of the time. Examples of stimuli used in Study 2 are shown in Fig 1D and 1E.

Similar to the analysis performed for Study 1, we calculated the absolute values for the similarity-confidence ratings and performed an ANOVA to determine if masked faces reduced reviewer similarity-confidence scores. Assigned condition significantly affected reviewer similarity-confidence ratings (*F*(2, 494) = 5.42, *p* = 0.0047). Specifically, reviewers in the control condition (no mask) had significantly higher similarity-confidence ratings (*μ* = 2.11) than reviewers in 65-algorithm condition (*p* = 0.011, *μ* = 1.97) and 95-confidence condition (*p* = 0.0022, *μ* = 1.94). There were no significant differences in similarity-confidence ratings for reviewers in the 65-algorithm and 95-algorithm conditions. By informing reviewers that there could be few or many mistakes made by an algorithm resulted in similar decreases in similarity-confidence ratings. These results demonstrate that masks in combination with algorithm information lead to decreases in similarity-confidence ratings whereas in Study 1, the presence of masks did not affect confidence ratings.

#### Algorithm accuracy rates do not impact reviewer accuracy

To examine the influence of presenting algorithm accuracy rates on reviewer performance, we computed accuracy metrics using the same decision threshold (*θ* = 0.5) as in Study 1. Table 2 shows accuracy rates, FPR, and TPR for each task condition. Overall, reviewers in the control condition (no masks present, no algorithm decisions present) had the highest levels of accuracy, 78% correct, nearly identical to the control condition accuracy rate observed in Study 1 (control condition accuracy rate = 77%) and our previous research [21]. Further, as expected from Study 1, masks reduced reviewer accuracy, however accuracy rates did not vary across other task conditions (Table 2).

**Table 2:** Face matching performance as a function of survey condition for Study 2 (threshold = 0.5).

Condition	Mask/No Mask	Algorithm Decision	n	% Correct (95% CI)	FPR (95% CI)	TPR (95% CI)	Sensitivity—*d’* (95% CI)	Criterion—*c* (95% CI)
65-Algorithm	Mask	Different	162	0.68 (0.64–0.71)	0.24 (0.20–0.28)	0.59 (0.54–0.64)	0.94 (0.76–1.12)	0.24 (0.15–0.33)
65-Algorithm	Mask	Same	162	0.69 (0.65–0.72)	0.29 (0.24–0.34)	0.66 (0.62–0.70)	0.97 (0.79–1.14)	0.08 (-0.02–0.17)
95-Algorithm	Mask	Different	172	0.70 (0.67–0.73)	0.21 (0.16–0.25)	0.61 (0.57–0.65)	1.09 (0.91–1.26)	0.27 (0.17–0.36)
95-Algorithm	Mask	Same	172	0.67 (0.64–0.70)	0.32 (0.27–0.37)	0.67 (0.63–0.70)	0.90 (0.74–1.07)	0.02 (-0.07–0.11)
Control	No Mask	None	163	0.78 (0.76–0.80)	0.17 (0.14–0.20)	0.72 (0.69–0.76)	1.54 (1.40–1.69)	0.18 (0.09–0.26)

Algorithm accuracy rates provided to reviewers did not alter their face matching accuracy. A repeated measures ANOVA found no significant effect of the presented algorithm accuracy rates on reviewer accuracy (*F*(1, 332) = 0.30, *p* = 0.59). As in Study 1, there was also no significant effect of algorithm decisions on accuracy (*F*(1, 332) = 0.26, *p* = 0.61) and no significant interaction between presented algorithm accuracy rates and algorithm decisions (*F*(1, 332) = 1.01, *p* = 0.32).

#### Algorithm accuracy rates decrease the influence of algorithm decisions

For each face pair in Study 2, we quantified shifts in average similarity-confidence responses as a function of algorithm decision (“SAME PERSON” or “DIFFERENT PEOPLE”) for each test condition (65-algorithm versus 95-algorithm) for each of the 12 face pairs individually. In the 65-algorithm condition, none of the shifts in similarity-confidence ratings were statistically significant, as compared with 5 of the 12 pairs reaching statistical significance in the 95-algorithm condition. Across the 12 pairs, similarity-confidence shifts were larger in the 95-algorithm condition relative to the 65-algorithm condition, but the effect did not reach statistical significance (*t*(11) = 2.14, *p* = 0.06).

**Fig 3:**
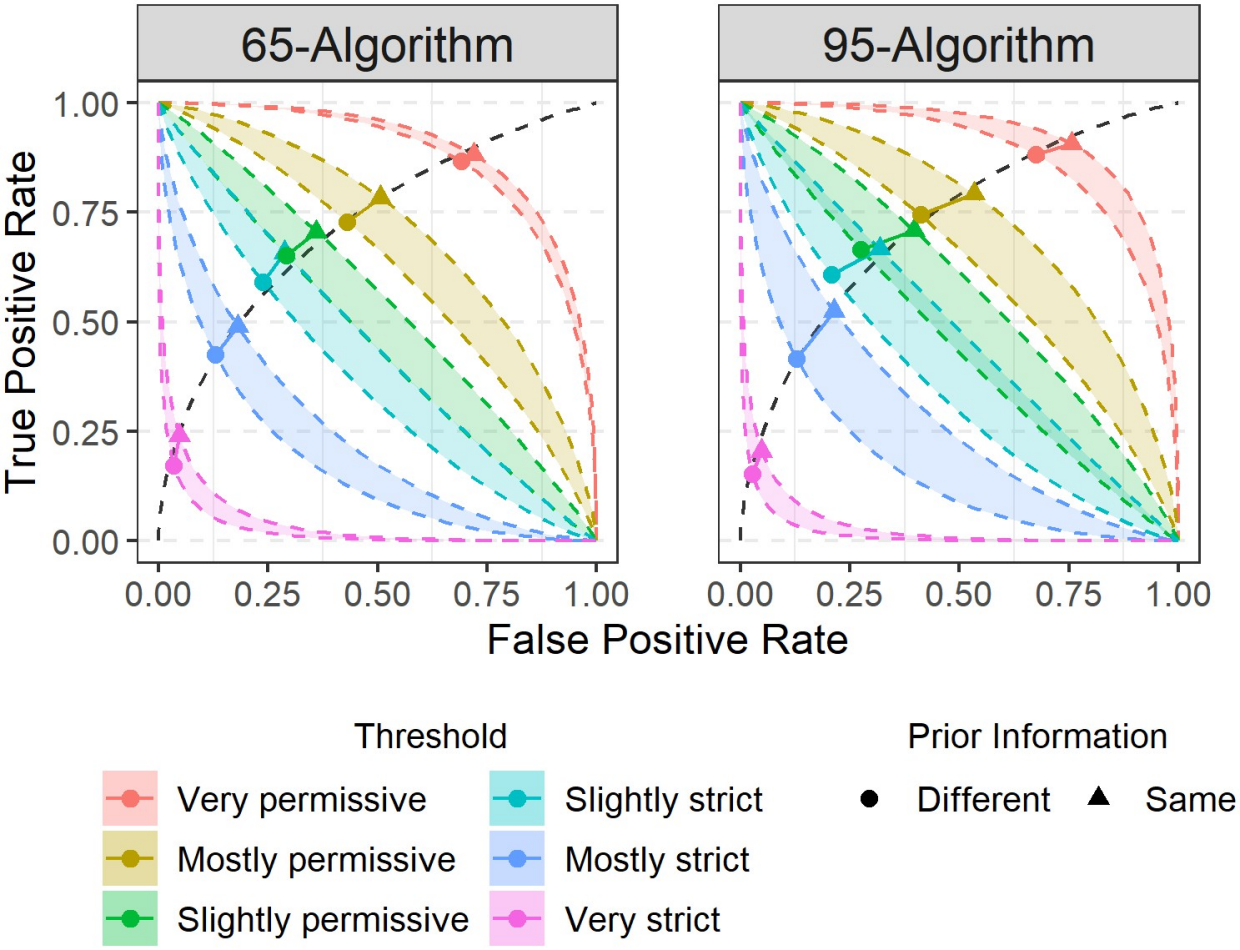
Shifts in responses for each algorithm condition. There is greater overlap of bands in the 95-algorithm condition compared to the 65-algorithm condition, demonstrating that the 95-algorithm influenced decisions to a greater degree than the 65-algorithm.

Reviewer cognitive biases observed in Study 2 were modest despite the presence of masks (Table 2). Reviewers assigned to the 65- and 95-algorithm conditions had smaller criterion shifts (65-algorithm criterion shift = 0.16, 95-algorithm criterion shift = 0.25). These shifts are a notably smaller than the 0.45 shift observed in Study 1 where algorithm accuracy rates were absent. This suggests that reviewer awareness of algorithm errors can markedly reduce the amount of cognitive bias during review. Cognitive bias for the 65-algorithm condition was lower than in the 95-algorithm condition. This indicates that reviewers may calibrate the degree of cognitive bias in their decisions based on information about algorithm accuracy rates. Fig 3 visualizes these results across reviewer decision thresholds, showing that isobias bands in the 95-algorithm condition were uniformly wider and overlapped more than in the 65-algorithm condition.

#### Reviewer perception of algorithms

While reviewers completed the face matching task, they did not receive any feedback whether their responses were correct or incorrect. We found that reviewers’ perception and trust of the algorithms in Study 2 differed based on the information presented at the beginning of the task. In Study 2, we asked reviewers if they trusted themselves or an algorithm to identify a person before and after the task (wording of the question per condition and responses options are documented in S1 File). Prior to starting the task, 77% of reviewers in the control condition indicated that they would trust another person to make identity decisions. When asked the same questions again after the task, reviewers in the control condition reduced their level of agreement to 68%. Reviewers in the experimental conditions altered their responses after the task based on the condition they were assigned to. At the beginning of the task, 69% of reviewers in both experimental conditions indicated they would trust an algorithm but by the end of the task, only 53% of reviewers in the 95-algorithm condition and 45% of reviewers in the 65-algorithm condition indicated they would trust an algorithm. After completing the trust question, we also asked reviewers to estimate how accurate they believed the algorithms and themselves were at the task (wording and responses options appear in S1 File). Despite the labels and instructions we provided in the task, the algorithms were only accurate 50% of the time in both experimental conditions. We found that reviewers generally believed the algorithm rates and selected responses that aligned with the initial accuracy rates. These results highlight that reviewers’ perception and trust in the algorithms were modulated by the initial information provided at the beginning of the task.

#### Analysis of cognitive bias across tasks

**Fig 4:**
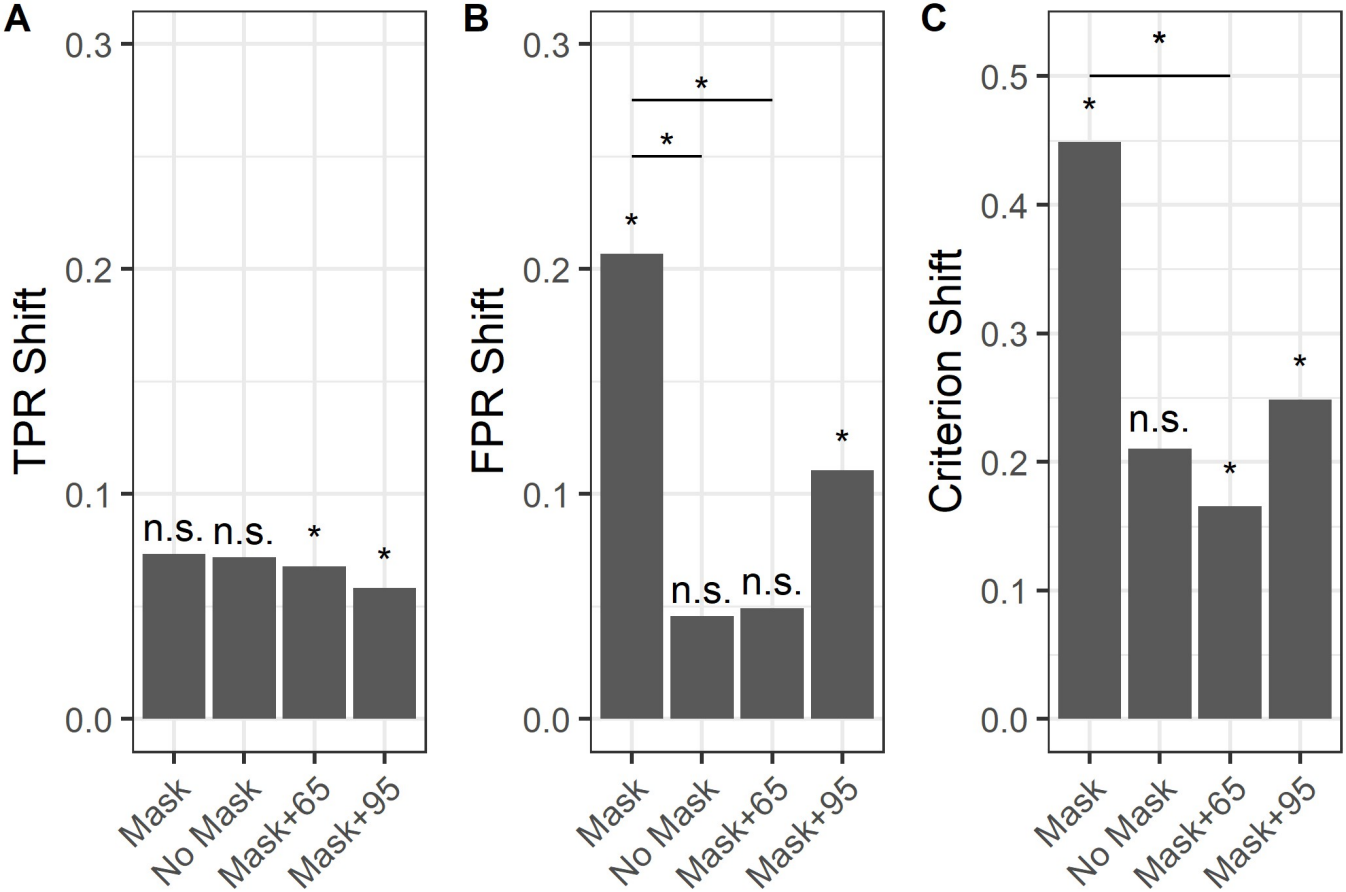
Panel A compares shifts in True Positive Rates (TPR) across Study 1 and 2 conditions. Panel B compares shifts in False Positive Rate (FPR) across Study 1 and 2 conditions. Panel C compares Criterion shifts across Study 1 and 2 conditions. The Mask and No Mask bars are the experimental conditions from Study 1. The Mask+65 and Mask+95 correspond to the 65- and 95-algorithm conditions in Study 2, respectively. Significant (*p* < 0.05) shifts within a condition are denoted by an asterisk (*) and non-significant shifts are indicated by n.s. over the bars. Significant (*p* < 0.05) shifts across conditions and studies are indicated by horizontal lines and asterisks (*).

We conducted a bootstrap analysis of data gathered across both studies to assess the combined influence of face masks and presented algorithm accuracy rates across all tested conditions (Fig 4). Specifically, we quantified the degree to which algorithm decision (“SAME PERSON” or “DIFFERENT PEOPLE”) shifted reviewer true positive rates (TPR), false positive rates (FPR) as well as the resulting shifts in the criterion (c). Shifts in true positive rates (Fig 4A) were not significant in Study 1, but were significant in each condition of Study 2, likely due to larger sample size. There were no significant differences in TPR shifts across conditions. Shifts in false positive rates (Fig 4B), however, were significant in the mask condition of study 1 as well as in the 95-algorithm condition of Study 2. Further, FPR shifts in the mask condition were significantly larger than FPR shifts in the no-mask condition of Study 1 and in the 65-algorithm condition of Study 2. Finally, criterion shifts (Fig 4C) were significant in the mask condition of Study 1 and in both conditions of Study 2. Criterion shifts in the 65-algorithm condition of Study 2 were the smallest and these were significantly smaller than criterion shifts observed in the mask condition of Study 1. Taken together, these results indicate that masks increase reviewers’ reliance on algorithm information, particularly on their likelihood of agreeing with a false positive decision and that this effect is mitigated by presenting information about the algorithms’ accuracy to the reviewers.

## Discussion

Human oversight of face recognition is currently used to prevent or mitigate risks associated with the use of these systems in high-risk applications, such as in security operations. However, there is a gap in data regarding the effectiveness of such practices [30]. Our prior research has found that human reviewers of face recognition algorithm decisions are cognitively biased by this prior information [21]. Here we find that this cognitive bias grows, apparently without reviewer awareness, even as the face information available to both humans and algorithms, decreases. Face masks reduced reviewer accuracy and increased their cognitive bias, but did not reduce their similarity-confidence ratings. That is, despite the increase in task difficulty and reliance of algorithm information, reviewers remained equally confident in their decisions. Prior research has shown that performance on standardized face matching tasks does not correlate to professional experience with unfamiliar face matching [31] and that subjective measures of face matching ability only modestly correlate with standard tests [32]. Our results indicate further that reviewers may not track the degree to which their review decisions are based on their own perceptual information versus face recognition algorithm decisions. This suggests that the safeguards offered by human oversight of face recognition technology may be limited, particularly in challenging environments. We demonstrate this using arbitrary algorithm decisions. To better understand these limitations, it will be necessary to directly evaluate human ability to detect actual errors produced by operational face recognition systems.

Our work suggests that reviewer training to raise awareness of algorithm errors may help reduce biases introduced by algorithm decisions in human review. In our study, providing reviewers with estimates of algorithm accuracy mitigated their increased reliance on algorithm decisions in the presence of face masks without affecting their accuracy. The high cognitive bias observed in the mask condition may indicate the fact that, by default, reviewers assumed that algorithms were highly accurate and deferred to automation in the face of increased task difficulty [33]. In our study, algorithm decisions biased reviewer responses even when they were told the algorithm was only 65% accurate, about as accurate as the human reviewers included in the study. Indeed, in Study 2, we observed a decrease reviewer trust in the algorithms after they completed the task, even in the absence of any feedback, indicating that reviewers updated their perception of algorithm accuracy by comparing their own perception to algorithm decisions. If there is a lack of trust in a system, then there is an increased likelihood that users will rely on their own judgment [34]. Given that humans perform poorly on unfamiliar face matching tasks [6], reviewer notions regarding algorithm accuracy formed based on comparisons of algorithm decisions to their own perception may be inaccurate. Such erroneous perceptions can arise, for instance, if pairs of faces that appear to be from the same person to reviewers are, correctly, judged as belonging to different people by algorithms. To calibrate reviewer trust such that algorithm decisions are properly integrated into face recognition workflows, reviewer training programs must foster a realistic understanding of algorithm performance relative to human capabilities.

The value of human review for face recognition algorithm decisions may need to be considered more carefully. Recent work has suggested that human review reduces errors in identity workflows relying on face recognition [19] and human review is recommended to “catch” face recognition algorithm errors in security applications [35–37]. However, the effectiveness with which human reviewers, based on their own perceptual faculties, could catch specific types of errors made by modern face recognition algorithms remains largely untested (but see [38]) and will likely change as the technology improves. Human review of algorithm decisions will remain fundamentally limited by the reviewers’ unfamiliar face matching ability. In our study, “reviewers” drawn from the general public caught our simulated false positives less than 80% of the time and caught false negatives less than 75% of the time, but none of the stimuli used in our study produced an error using modern commercial face recognition algorithms.

Indeed false positives made by current commercial face recognition algorithms configured for security would likely be infrequent and much harder for humans to detect [38] as they would likely be between two individuals sharing perceptually salient characteristics [39, 40]. Indeed, current commercial face recognition algorithms are frequently evaluated with false positive error rates set to just one in a million comparisons [41]. Prior work suggesting that human-algorithm teams produce optimal performance did not separately consider false positives and false negatives, but likely relied primarily on the ability of humans to overcome algorithmic false negatives [19]. False negatives can occur due to factors unrelated to face characteristics, such as poor image quality or due to factors that may be easier for a human to detect, such as the presence of an injury, occlusions due to a face mask, or makeup. For example, recent work shows that reductions in algorithm matching performance in the presence of face masks are due to increases in false negatives, not false positives [42]. A human reviewer could easily identify the presence of a face mask, but may have great difficulty adjudicating an algorithmic false positive. Future work should examine the effectiveness of human oversight in detecting algorithmic false positives and false negatives separately. This will help understand the likely effectiveness of human review in the different use-cases of face recognition technology and to determine those for which human perceptual review of the faces is meaningful and those for which reviewers should focus on information other than facial features.

Our work focuses on evaluating human behavior in human-algorithm teaming; developing novel algorithms is beyond the scope of the present work. We encourage face recognition algorithm developers to consider alternate methods of algorithm optimization when the algorithms are to be deployed in a setting with a human teammate. Current applications of biometric algorithms rely on humans to compensate for the weaknesses of an algorithm, which are often the same weaknesses of human face matching ability. Creating algorithms that make unique mistakes from humans or leverage the strengths of the human operator may help ensure that the biometric system and team performance is enhanced [43]. Future work should compare the performance of human-algorithm teams with different algorithm teammates that have alternate methods of optimization.

Limitations of our studies include mask application, a lack of diversity of face pairs, and the frequency of algorithm errors. The masks on our stimuli were digitally applied and do not fully capture the effects masks may have on face matching. Despite only emulating masks, we believe that the masks we used are fair representations of general mask usage covering similar portions of the face as NIST evaluations [25] and other recent research [24, 26]. Another limitation of our studies is the diversity of stimuli. The GFMT consists of primarily young, Caucasian adults; we included face stimuli from MEDS to be similar to the demographics of our sample, however we were unable to include other minorities as there are very few datasets that match the controlled style of the GFMT dataset. An additional limitation of our study was the error rate of algorithms; we set our algorithm to a 50% accuracy level but this is not representative of the frequency of algorithm errors observed in real-world scenarios. In many applications, accuracy rates can be in excess of 95% with many algorithms achieving even higher rates [13]. Future research should investigate the types of errors automated face recognition algorithms make at the incidence they occur and determine the frequency in which human operators can identify these errors.

## Supporting information

S1 File Supplemental information with details about the methods for each study, additional analyses, and results are described in this document. (DOCX)

S2 File Supplemental information with task data from study 1. (CSV)

S3 File Supplemental information with trust data from study 1. (CSV)

S4 File Supplemental information with task data from study 2. (CSV)

S5 File Supplemental information with trust data from study 2. (CSV)
