# Supplementary material for: COVID-19 masks increase the influence of face recognition algorithm decisions on human decisions in unfamiliar face matching
Source: PLoS One. 2022 Nov 21;17(11):e0277625. doi: 10.1371/journal.pone.0277625 (PMC9678274; doi:10.1371/journal.pone.0277625)
Supplement: S1 File — (DOCX) [file pone.0277625.s001.docx]

**COVID-19 masks increase the influence of face recognition algorithm decisions on human decisions in unfamiliar face matching**

Supplementary Material

**SUPPLEMENTARY METHODS**

**Digital Masks**

All image manipulations were done using the Python programming language. Masks were applied digitally using face points generated by the Dlib image processing library (Ngan, Grother, & Hanaoka, 2020). The Dlib library included a face detector and a standard 68-point facial landmark detector. The face mask region was drawn as a closed polygon using face points 3 and 15 below the ears, 30 on the bridge of the nose and all points along the chin between points 3 and 15. Once the mask polygon was defined, image pixels within the masked area were filled with neutral gray (hexacode #808080).

**Trust Question**

At the beginning of the task, reviewers were asked a multiple-choice question regarding their trust in their ability or the ability of a computer to identify a person. Reviewers assigned to the control condition were asked: Would you trust yourself to identify a person? Reviewers assigned to the other two conditions were asked: Would you trust a computer to identify a person? Reviewers were to select one of the following response options: *no*, *not sure*, or *yes*. This question was used to assess trust in the source that reviewed each face pair. No follow-up questions regarding trust were asked at the end of the survey to match prior research (Howard, Rabbitt, & Sirotin, 2020).

**Face Matching Task**

The face matching task administered as part of our study used the same base face pairs as Howard et al. (2020) and included face pairs from the short version of the GFMT (Glasgow Face Matching Test; Megreya & Burton, 2006), face pairs from the NIST Multiple Encounters Dataset (MEDS; Founds, Orlans, Genevieve, & Watson, 2011), and two easy to recognize celebrity face pairs (for details see Howard et al., 2020). The celebrity face pairs were well known, U.S. public figures, likely to be familiar to our test population recruited from the local area, and therefore easy to distinguish and match (Megreya & Burton, 2006). All face pair stimuli were presented to all reviewers in the same order regardless of experimental condition.

**Response Times**

Response times were measured as the last click on the page before pressing the button to move to the subsequent question. Response times were measured in seconds. Response times were visually inspected to identify outliers; a threshold of 2-standard deviations above the mean was applied to remove extremely long response times from the analysis. This threshold was applied to each condition, rather than defining a cut-off point to prevent substantial exclusion of data from the analysis (5% of data was omitted from analysis with the exclusion criteria). Following outlier removal, mean item-response times were computed for each condition in Study 1 and Study 2.

**DATA ANALYSIS**

**Item-Level Analysis**

To determine the degree to which algorithm information influenced responses in each condition, we conducted an item-level analysis. To do this, we examined the average responses for each face pair for each algorithm decision and then subtracted the average responses for “SAME PERSON” from “DIFFERENT PEOPLE”. The average of those differences were then compared using a Wilcoxon test to determine if the shift was significant based on algorithm decision for a given face pair between two study conditions. To determine if these shifts were greater in one condition compared to another, the average differences for each condition were compared using a t-test.

**Accuracy and Error Rates**

To calculate true positive rate (TPR_θ_), false positive rate (FPR_θ_), and overall accuracy (ACC_θ_) we converted certainty responses for each face pair *R_i_* to binary decisions (1=same, 0=different) using a sliding threshold θ ϵ (-2.5, -1.5, -0.5, 0.5, 1.5, 2.5), described in **Table S 1**.

| *θ* | Description |
| --- | --- |
| 2.5 | Very strict |
| 1.5 | Mostly strict |
| 0.5 | Slightly strict |
| -0.5 | Slightly permissive |
| -1.5 | Mostly permissive |
| -2.5 | Very permissive |

**Table S 1. Threshold values to calculate TPR and FPR.**

For each threshold value, we calculated the true positive rate TPR_θ_, the false positive rate FPR_θ_, and overall accuracy ACC_θ_ using the following formulas:

$${TPR}_{\theta}=\frac{1}{n}\sum_{same} R_{i} > \theta$$

$${FPR}_{\theta}=\frac{1}{m}\sum_{different} R_{i} > \theta$$

$${ACC}_{\theta}=\frac{n\left( {TPR}_{\theta} \right)+m\left( 1- {FPR}_{\theta} \right)}{n+m}$$

**SIGNAL DETECTION THEORY**

In all experimental conditions, we examined the effects of an algorithm decision in the context of signal detection theory, which separates reviewers' sensitivity to face similarity from any cognitive bias in making same/different judgments. We first measured changes in an observer's sensitivity, i.e., a change in how well an observer can discriminate faces of the same individual from those of different individuals. A drop in sensitivity would be expected if adding information diverts spatial attention away from the face stimuli. Sensitivity is measured by the dʹ metric. The second means of impacting cognitive tasks is by raising or lowering the internal criterion, i.e., the required similarity to judge a pair of faces as being of the same individual.

**Quantifying Shifts in Error Rates and Bias**

We examined how True Positive Rates (TPR), False Positive Rates (FPR), and criterion (c) values differed across all conditions by examining the difference in responses based on algorithm decisions. To determine the differences between TPR and FPR, the values associated with algorithm “DIFFERENT PEOPLE” decisions were subtracted from algorithm “SAME PERSON” decisions. We ran a two-tailed t-test to determine if these differences were significant. To determine if there were significant differences in criterion values, we ran bootstrap analysis with 10,000 replications to estimate criterion values for each experimental study condition.

**STUDY 1**

*Reviewers*

A total of 153 reviewers were recruited from the local Washington D.C., Virginia, and Maryland area for a biometrics test that occurred in August 2020. After the biometrics test, reviewers completed the face matching task. **Table S 2** shows the age and gender demographics of reviewers assigned to each survey condition. Reviewer assignment to each survey condition was randomly determined by the survey software, Qualtrics (Provo, UT, USA), administered on iPads (Apple; Cupertino, CA). A total of 3 reviewers failed to correctly identify both celebrity face pairs and were excluded from analysis leaving a total of 150 reviewers whose data was analyzed.

| Condition | Mean (SD) Age | Gender | | | N |
| --- | --- | --- | --- | --- | --- |
|  |  | Female | Male | Missing |  |
| Computer – No Mask | 39.10 (12.57) | 30 | 19 | 2 | 51 |
| Computer – Mask | 43.73 (13.45) | 20 | 29 | 1 | 50 |
| Control | 43.21 (14.32) | 24 | 23 | 2 | 49 |

**Table S 2. Study 1 reviewer demographics by condition.**

*Experimental Conditions*

Reviewers were randomly assigned to one of three conditions including a control condition and two experimental conditions: no mask and mask. The mask condition included one face wearing a mask to emulate real-world conditions where a reviewer would match a masked face image to a non-masked reference images, for example from a travel document (Section *Digital Masks under Supplementary Methods*).

No face masks appeared in the no mask condition. In the mask condition, the face on the right appeared with a digitally applied face mask. The face matching task followed a nested design as in Howard et al. (2020) where there were two versions of each experimental condition. Algorithm decision was nested within computer and mask conditions and indicated per face pair using a colored background and a text label (Told Same: green background, “SAME PERSON” text underneath face pair; Told Different: orange background, “DIFFERENT PEOPLE” text underneath face pair). The control condition presented each face pair with a grey background with the instruction “COMPARE FACES” underneath each face pair. An algorithm decision was presented simultaneously to the reviewers with each face pair. Two versions of each survey with algorithm decisions ensured that each face pair was presented under both same and different instructions to half of the reviewers in each group.

**STUDY 2**

*Reviewers*

Reviewers were recruited from the local Washington D.C., Virginia, and Maryland for a biometrics test that occurred in September and October 2021. After reviewers completed the biometrics tests, reviewers completed the face matching task. **Table S 3** shows the age and gender demographics of reviewers assigned to each survey condition. Reviewers assignment to each survey condition was randomly determined by the survey software, Qualtrics (Provo, UT, USA), administered on iPads.

| Condition | Mean (SD) Age | Gender | | N |
| --- | --- | --- | --- | --- |
|  |  | Female | Male |  |
| 65-Algorithm | 47.73 (14.82) | 91 | 71 | 162 |
| 95-Algorithm | 46.99 (13.86) | 96 | 76 | 172 |
| Control | 47.60 (14.97) | 86 | 77 | 163 |

**Table S 3. Study 2 reviewer demographics by condition.**

*Experimental Conditions*

Reviewers were randomly assigned to one of three conditions including a control condition and two experimental conditions: 65-algorithm and 95-algorithm. Both experimental conditions included a digitally applied face mask similar to the mask-condition of Study 1. In both conditions, reviewers were given information about algorithm accuracy prior to starting the face matching task. In the 65-algorithm condition reviewers were told that the computer that reviewed the face was accurate 65% of the time. In the 95-algorithm condition reviewers were told that the computer that reviewed the face was accurate 95% of the time.

At the beginning of the task, users were provided the following instructions:

For the next few questions, we are going to show you pairs of faces. A computer has examined each pair of faces and decided if the faces are the same person or different people. We think the computer's decisions are correct XX% of the time. Your task is to determine if the face pair shows the same person or shows different people. The decision for each face pair may be different, so please read each question carefully!

The XX% text was filled with either 65% or 95% depending on the condition the reviewer was assigned to. If the reviewer was assigned to the control condition, the instructions were abbreviated and omitted the information about algorithm accuracy and reviewing decisions for each face pair. Reviewers assigned to the 65- and 95-algorithm conditions were also shown a large computer icon with the accuracy rate displayed on the screen. Additionally, each face pair had a computer icon with the accuracy rate displayed beneath each face pair as a reminder during the task.

To ensure that reviewers understood the accuracy of the algorithm, we inserted a question where reviewers were to type the numerical value of the accuracy rate without the percentage sign. Reviewers could not proceed to the task without a correct response. If a reviewer had difficulty with this question, they raised their hand and a test staff would verbally explain the question and intent. The test staff would then press the back button to go to the instruction page and review the instructions verbally with the reviewer. No reviewers failed to understand the task after the verbal explanation.

*Pre- and Post-Task Trust/Accuracy Assessment*

Reviewer trust was assessed before and after the face matching task. Reviewers were asked the trust question (see Section *Trust Question under Supplementary Methods*) before starting the face matching task and again after they completed the task. Reviewers in experimental conditions were asked about their degree of trust with a computer to identify a person, and reviewers assigned to the control condition were asked about their degree of trust in themselves to identify a person. In Study 1, reviewers were only asked this question at the beginning of the study. To determine if reviewers’ level of trust changes after reviewing the accuracy of algorithm decision for each face pair, we asked this question again after the face matching task.

At the end of the task, reviewers were asked one of the following accuracy questions:

How do you think the computer was correct during the task? (65- and 95- conditions)

How often do you think you were correct during the task? (control condition)

Reviewers were asked to select one of the following response options: Correct 89% - 100% of the time, Correct 76% - 88% of the time, Correct 63% - 75% of the time, 50% - 62% of the time, Correct less than 50% of the time.

**SUPPLEMENTARY RESULTS**

**Study 1**

*Trust Question*

**Figure S 1** shows the proportion of responses per condition to the trust question: Do you trust X to make identity decisions? X was the condition assigned the reviewer was assigned to. If the reviewer was assigned to the control condition, X was yourself. If the reviewer was assigned to the experimental condition, X was a computer. Paid volunteers responded to the trust question with the following response options: *Yes, No, Not Sure.* As can be seen in the figure, reviewers in the control condition had the highest proportion of trust before completing the face matching task. A chi-squared test was conducted to determine if there were significant differences in the way reviewers responded to the trust question across conditions and found that there were no differences (χ^2^ (2) = 4.53, *p* = 0.10). This indicates that for the trust question, reviewers' responses were similar regardless of which source (computer or self) reviewed each face pair. These results replicate the findings of our previous work, where there were not differing levels of trust between themselves and computers (Howard et al., 2020).


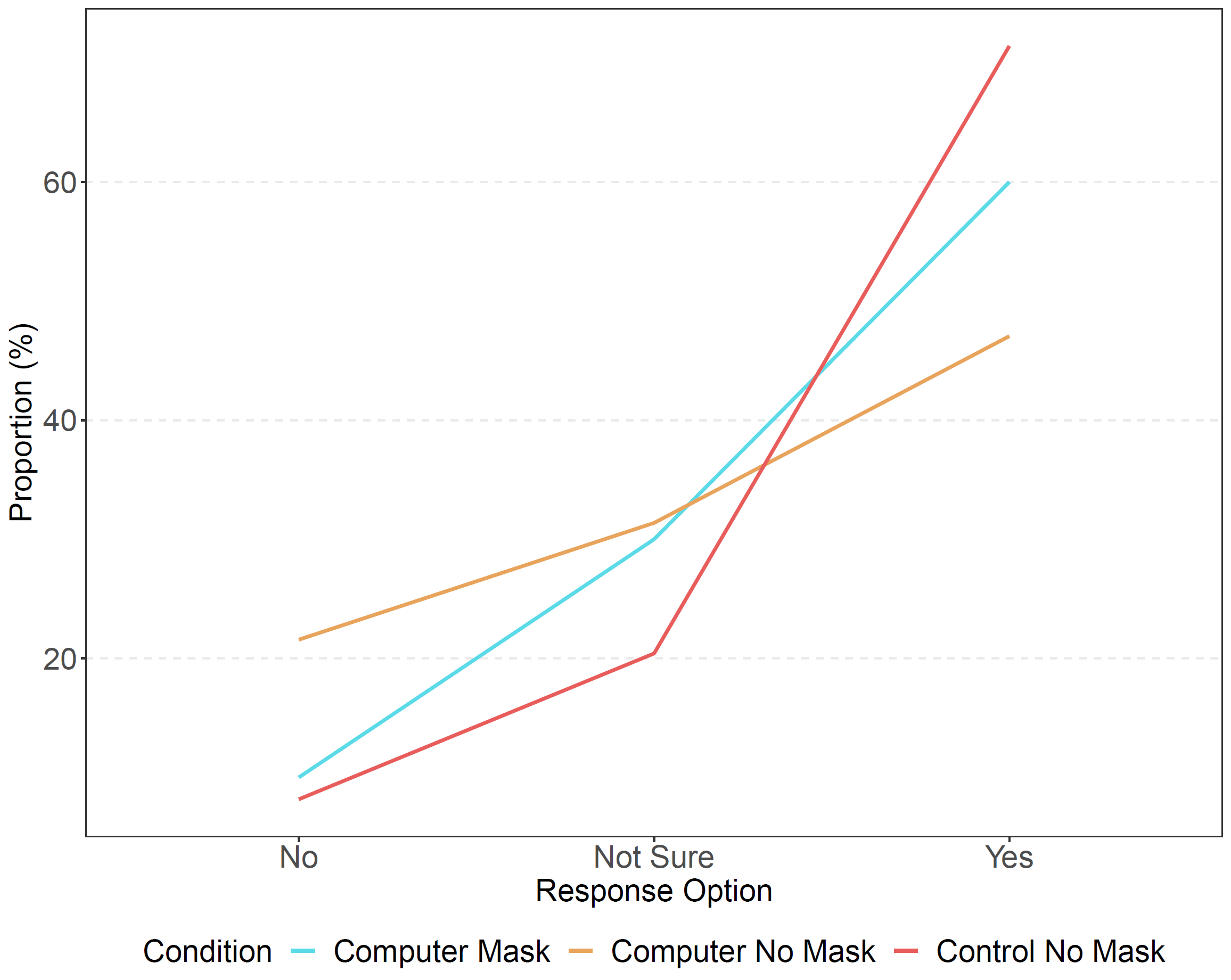


**Figure S 1. Trust scores per condition.**

*Item-level Analysis*

We examined the degree to which similarity-confidence ratings shifted for face pairs based on algorithm decision (“SAME PERSON” or “DIFFERENT PEOPLE”) in the mask and no mask conditions. **Figure S 2** shows how similarity-confidence ratings shifted for each face pair. Each point represents a single face pair. Significant shifts in similarity-confidence ratings in the mask condition are indicated by a filled circle. Significant shifts in similarity-confidence ratings based on algorithm decisions in both the mask and no mask conditions are indicated by a filled square. We conducted a t-test to determine if the algorithm decisions consistently shifted responses for the mask condition compared to the no mask condition using a t-test. This test was significant (*t*(11) = 2.63, *p* < 0.05) indicating that response shifts were bigger when masks were present.


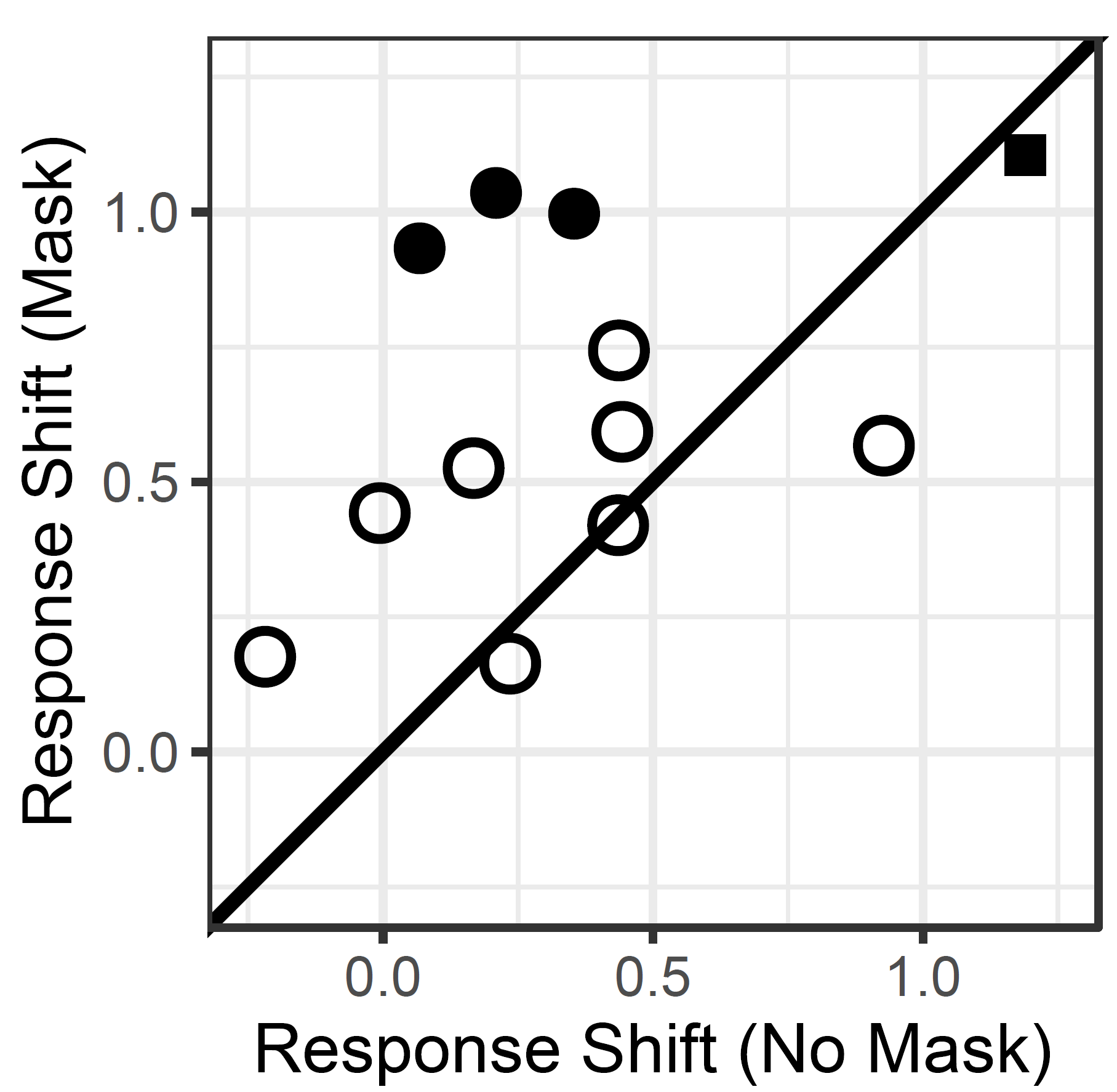


**Figure S 2. Shifts in similarity-confidence responses for each face pair in the mask and no mask conditions in Study 1. Each point represents a face pair; significant shifts for a given face pair are indicated by filled circles. Significant shifts in both mask and no mask conditions are indicated by a filled square.**

It is important to note that there were similar responses ranges in both the mask and no mask conditions. That is, reviewers in these two groups used the full range of the similarity-confidence rating scale (from -3 to 3). However, item responses shifted to a greater degree when masks were present than when masks were absent. These shifts were stable across all 12 face pairs.

**Study 2**

*Pre-Post Trust Assessment*

**Figure S 3** shows pre- and post-trust scores per condition. Reviewers in the control condition had the highest proportion of trust before and after completing the face matching task whereas, reviewers in the 65- and 95-algorithm conditions were less optimistic post-task. To determine if there were differences in trust question responses, we ran chi-square tests to determine if there were differences in responses between conditions before and after the task. We found that prior to beginning the task, there were not significant differences in reviewers' responses across conditions (χ^2^(4) = 5.71, *p* = 0.22). However, trust responses differed after the task, as indicated by significant differences in reviewers' responses across the conditions (χ^2^ (4) = 17.96, *p* < 0.005). Specifically, adjusted pairwise comparisons showed that reviewers in the control condition had significantly greater trust than reviewers in the 65- (*p* < 0.005) and 95-algorithm (*p* = 0.035) conditions. There were no significant differences between the 65- and 95-algorithm conditions (*p* = 0.30). Our results demonstrate how trust may change after a task, particularly based on informing users that algorithms can make mistakes.


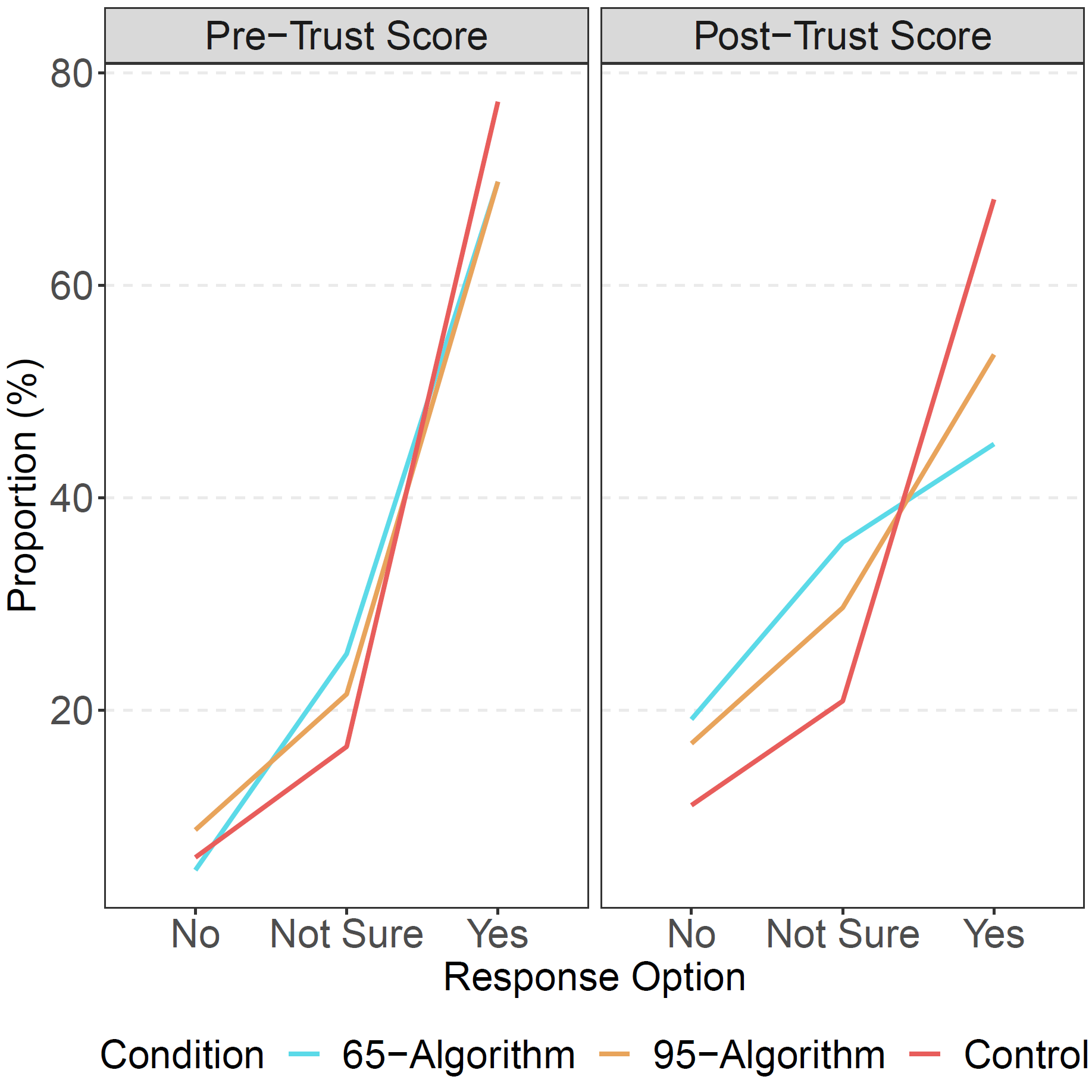


**Figure S 3. Pre- and post-trust scores per condition.**

*Perception of Algorithm Accuracy*

At the end of the survey, reviewers in the experimental conditions were asked to indicate how accurate they thought the algorithm was on the task. Reviewers in the control condition were asked to indicate how accurate they thought they were on the task. **Figure S 4** shows reviewers perceived accuracy of the system or themselves, depending on their condition. In the control condition, reviewers most reported that they perceived they were accurate 76-88% of the time. Reviewers in the 65-algorithm condition tended to select that the algorithm was correct 63-75% of the time, which was the range that corresponded to the initial algorithm information. Reviewers in the 95-algorithm condition selected the 89-100% or 76-88% accuracy rate options, either similar to or slightly lower than the initial algorithm information. These options highlight that reviewers generally believed the algorithm rates they were told at the beginning of the survey. However, the algorithms were only 50% accurate.


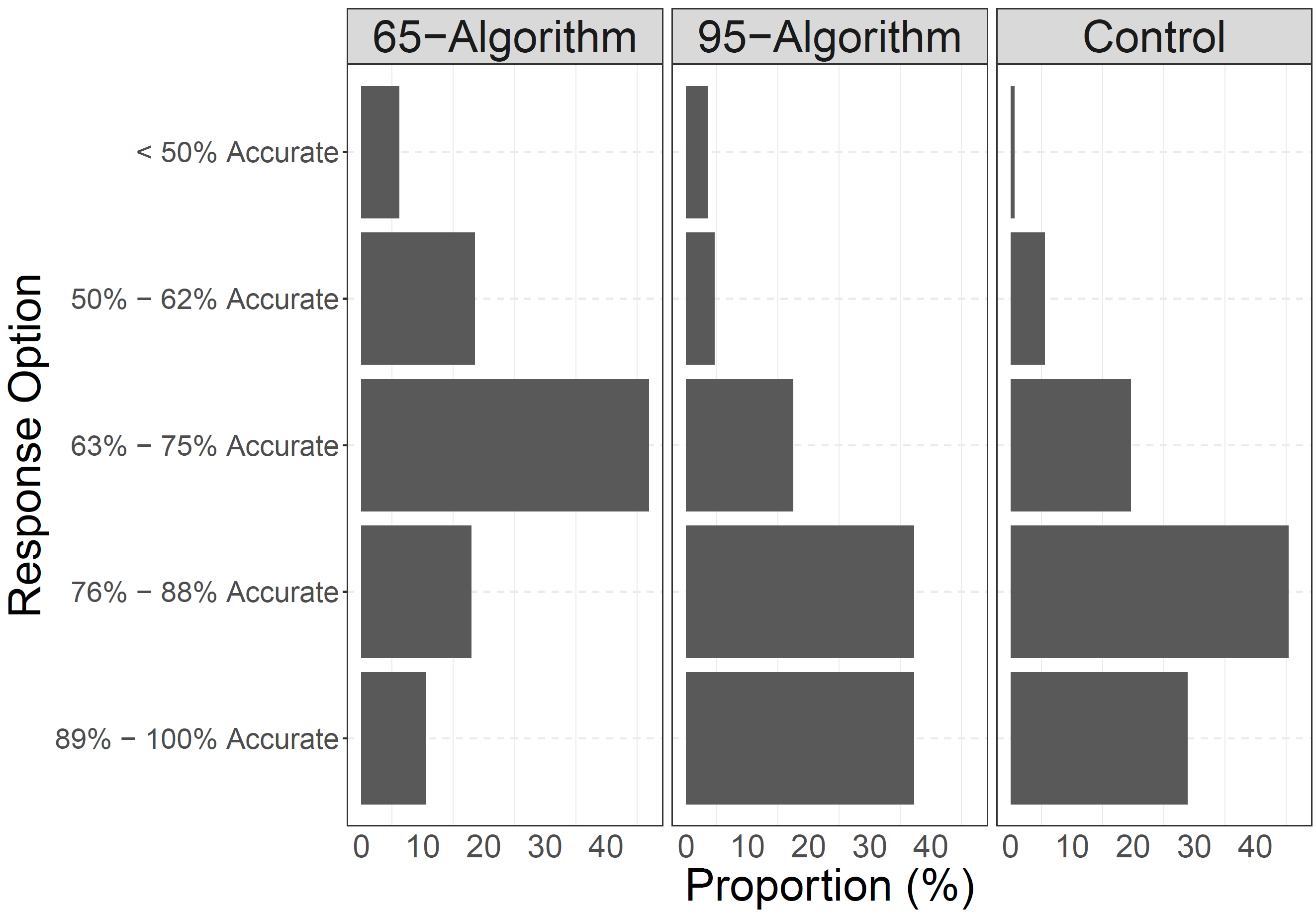


**Figure S 4. Perceived accuracy per condition.**

*Item-level Analysis*

We examined the degree to which similarity-confidence ratings shifted for face pairs based on algorithm decision (“SAME PERSON” or “DIFFERENT PEOPLE”) in the 65- and 95-algorithm conditions. **Figure S 5** shows how similarity-confidence ratings shifted for each face pair. Each point on the plot represents a face pair. Significant shifts in similarity-confidence ratings filled in circles represent significant shifts in distributions for the 95-algorithm condition. We performed a t-test to determine if these shifts were significant across all face pairs in the 95-algorithm condition over the 65-algorithm condition and found that this effect was trending significance (*t*(11) = 2.14, *p* = 0.06). These results show that there weren't differences of algorithm decisions across the algorithm accuracy conditions.


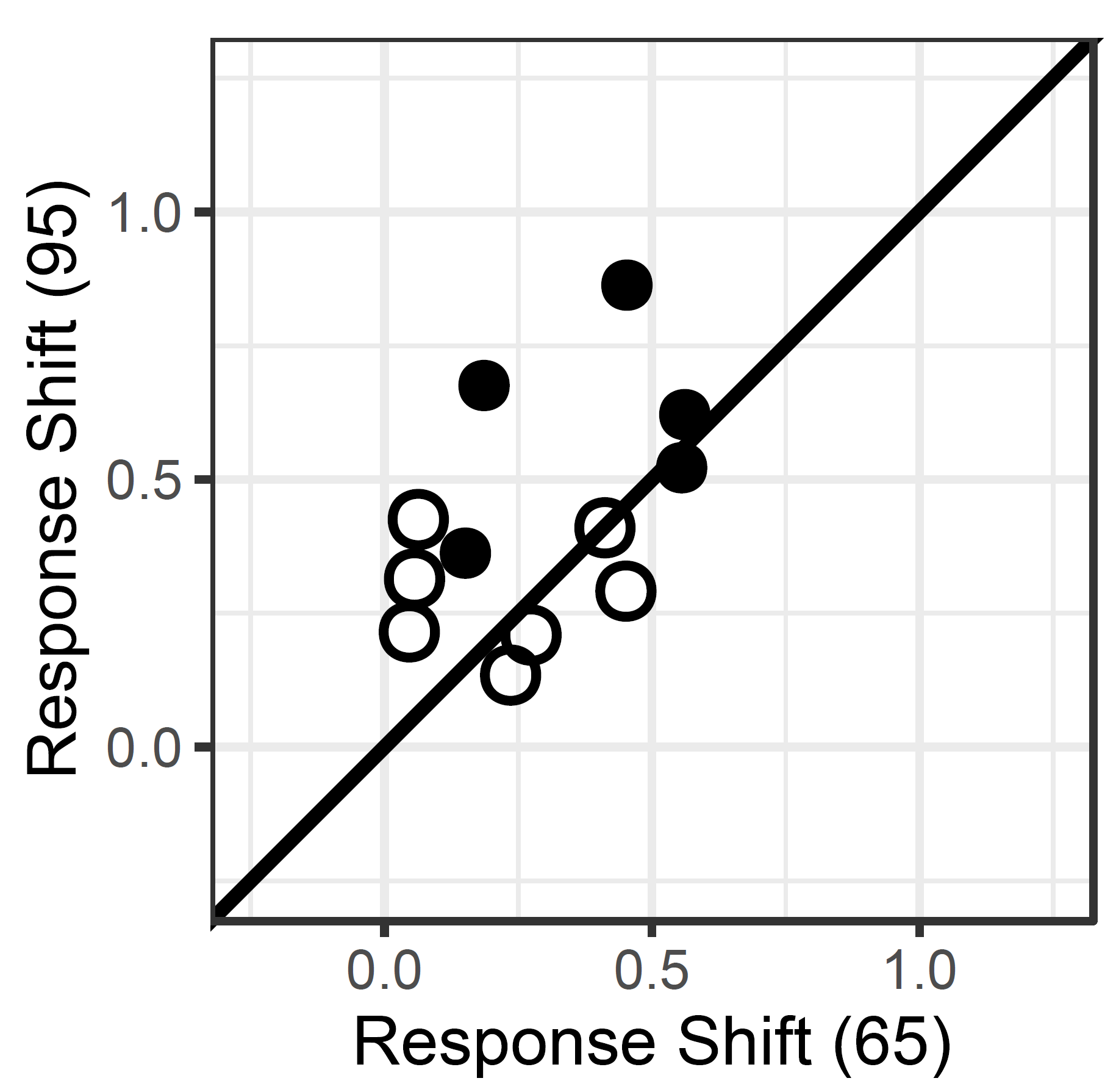


**Figure S 5. Shifts in similarity-confidence responses for each face pair in the 65- and 95-algorithm conditions in Study 2. Each point represents a face pair; significant shifts for a given face pair are indicated by filled circles.**

**Study 1 and 2 Comparison**

*Item-level Analysis*

We also examined the degree to which similarity-confidence ratings shifted for face pairs based on algorithm decision (“SAME PERSON” or “DIFFERENT PEOPLE”) in the mask condition from Study 1 and the 65-algorithm condition from Study 2. Points in **Figure S 6** represent a face pair; significant shifts in responses based on algorithm decisions for the mask condition in Study 1 are indicated by filled circles. We performed a t-test to determine if these shifts were significant across all face pairs for the mask condition from Study 1 over the 65-algorithm condition and found that this effect was significant (*t*(11) = 2.89, *p* < 0.05). These results demonstrate how providing information about an algorithm's performance can mitigate some shifts caused by masks.


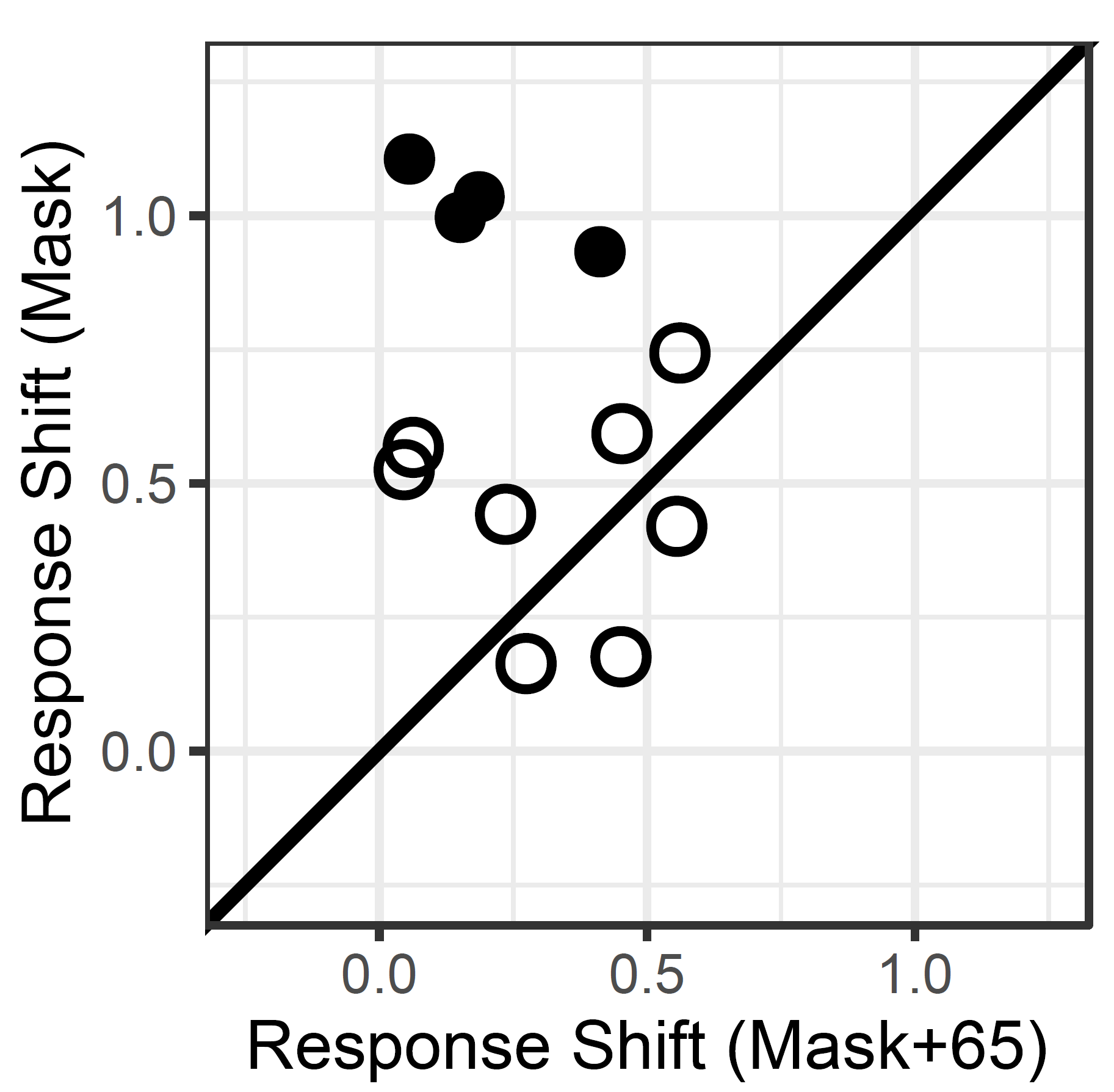


**Figure S 6. Response shifts for each face pair across the Mask condition in Study 1 and the 65-algorithm condition in Study 2. Each dot represents a face pair; significant shifts for a given face pair are indicated by filled circles.**

**SUPPLEMENTARY BIBLIOGRAPHY**

Burton, A. M., White, D., & McNeill, A. (2010). The Glasgow face matching test. *Behavior research methods*, *42*(1), 286-291.

Founds, A. P., Orlans, N., Genevieve, W., & Watson, C. I. (2011). NIST special database 32-multiple encounter dataset II (MEDS-II).

Howard, J. J., Rabbitt, L. R., & Sirotin, Y. B. (2020). Human-algorithm teaming in face recognition: How algorithm outcomes cognitively bias human decision-making. *Plos one*, *15*(8), e0237855.

Megreya, A. M., & Burton, A. M. (2006). Unfamiliar faces are not faces: Evidence from a matching task. *Memory & cognition*, *34*(4), 865-876.

Ngan, M. L., Grother, P. J., & Hanaoka, K. K. (2020). Ongoing Face Recognition Vendor Test (FRVT) Part 6A: Face recognition accuracy with masks using pre-COVID-19 algorithms.
